# Genotype-phenotype correlation in 22q11.2 deletion syndrome

**DOI:** 10.1186/1471-2350-13-122

**Published:** 2012-12-17

**Authors:** Elena Michaelovsky, Amos Frisch, Miri Carmel, Miriam Patya, Omer Zarchi, Tamar Green, Lina Basel-Vanagaite, Abraham Weizman, Doron Gothelf

**Affiliations:** 1 Sackler Faculty of Medicine, Tel Aviv University, Tel Aviv, Israel; 2 Felsenstein Medical Research Center, Petah Tikva, Israel; 3 The Child Psychiatry Unit, Edmond and Lily Safra Children’s Hospital, Sheba Medical Center, Tel Hashomer, Ramat Gan, Israel; 4 Nes-Ziyyona-Beer Yaakov Mental Health Center, Beer Yaakov, Israel; 5 Pediatric Genetics Schneider Children’s Medical Center of Israel and Raphael Recanati Genetics Institute, Rabin Medical Center, Petah Tikva, Israel; 6 Geha Mental-Health Center, Petah Tikva, Israel; 7 Felsenstein Medical Research Center (FMRC), Sackler Faculty of Medicine, Tel Aviv University, Rabin Medical Center, 49100, Petah Tikva, Israel

**Keywords:** Velocardiofacial syndrome (VCFS), Multiplex ligation probe amplification (MLPA), Copy number variation (CNV), Molecular diagnosis, Neuropsychiatric disorders

## Abstract

**Background:**

The 22q11.2 deletion syndrome (22q11.2DS) is caused by hemizygous microdeletions on chromosome 22q11.2 with highly variable physical and neuropsychiatric manifestations. We explored the genotype-phenotype relationship in a relatively large 22q11.2DS cohort treated and monitored in our clinic using comprehensive clinical evaluation and detailed molecular characterization of the deletion.

**Methods:**

Molecular analyses in 142 subjects with 22q11.2DS features were performed by FISH and MLPA methods. Participants underwent clinical assessment of physical symptoms and structured psychiatric and cognitive evaluation.

**Results:**

Deletions were found in 110 individuals including one with an atypical nested distal deletion which was missed by the FISH test. Most subjects (88.2%) carried the 3Mb typically deleted region and 11.8% carried 4 types of deletions differing in size and location. No statistically significant genotype-phenotype correlations were found between deletion type and clinical data although some differences in hypocalcemia and cardiovascular anomalies were noted.

Analysis of the patient with the distal nested deletion suggested a redundancy of genes causing the physical and neuropsychiatric phenotype in 22q11.2DS and indicating that the psychiatric and cognitive trajectories may be governed by different genes.

**Conclusions:**

MLPA is a useful and affordable molecular method combining accurate diagnosis and detailed deletion characterization. Variations in deletion type and clinical manifestations impede the detection of significant differences in samples of moderate size, but analysis of individuals with unique deletions may provide insight into the underlying biological mechanisms.

Future genotype-phenotype studies should involve large multicenter collaborations employing uniform clinical standards and high-resolution molecular methods.

## Background

The 22q11.2 deletion syndrome (22q11.2DS), also known as velocardiofacial syndrome (VCFS) and DiGeorge syndrome, is a genetic disorder caused by hemizygous microdeletions on chromosome 22q11.2, with population prevalence of about 1 to 4,000 births [1]. The 22q11.2DS has multisystem manifestations including conotruncal congenital heart defects (CHD), velopharyngeal anomalies, hypoparathyroidism, T-cell immunodeficiency, craniofacial features, cognitive deficits and high rates of psychiatric morbidity [e.g., schizophrenia, anxiety disorders and attention deficit hyperactivity disorder (ADHD)] [2-4]. The clinical phenotype is characterized by variable expression and incomplete penetrance.

The mechanism mediating 22q11.2 deletions involves non-allelic homologous recombination of low copy repeats (LCR) in 22q11.2. The majority (90%) of the 22q11.2 deletions are of 3Mb (encompassing ~60 genes) occurring between LCR A and LCR D and often referred to as typically deleted region (TDR) while 8% are 1.5Mb in size (~28 genes) (LCR A-B). Some rare atypical deletions of shorter size and in variable locations have also been reported [5]. We have long been interested in 22q11.2DS as a model for studying the genetic contribution to psychiatric disorders and for exploring the correlation between the molecular characteristics of the deletion and the cognitive and psychiatric manifestations in the patients.

The classic molecular diagnostic procedure used for detection of deletions and duplications at 22q11.2 is chromosomal analysis coupled with fluorescent *in situ* hybridization (FISH) using a single fluorescent probe (N25 or TUPLE1) located in the proximal part of the typically deleted region. The FISH technique which is still in routine use in many laboratories is unable to detect deletions that are either proximal or distal to the particular FISH probe used. In recent years several new methods for detecting the 22q11.2 deletion have been developed: comparative genomic hybridization (CGH) [6,7], multiplex ligation probe amplification (MLPA) [8,9], multiplex quantitative real-time PCR [10,11] and high-resolution SNP microarray analysis [10,12]. The above-mentioned whole-genome methods are still at the research stage and require expensive equipment for performing the assay and analyzing the data. The MLPA method uses multiple probes to achieve a good resolution combined with the practicality and affordability of a commercial kit, which is rapidly and easily performed in any experienced laboratory.

We evaluated the utility of MLPA as an alternative to FISH in diagnosing 142 Israeli individuals who had been referred to our clinic with the initial suspicion of 22q11.2DS and also as a tool for characterizing the deletion size and location. The information of the size and location of the deletion in the 22q11.2DS subjects is hereby presented together with their physical and psychiatric clinical features. This may help in the search for a possible genotype-phenotype correlation of different deletion types. The clinical phenotype of individuals with the typically deleted region and other less common deletions are compared by quantitative measures and also by descriptive means. In addition, the physical and longitudinal neuropsychiatric phenotype of an individual with a rare short deletion is described in detail.

## Methods

### Subjects

The study included 142 individuals referred to the Behavioral Neurogenetics Center (BNC) at Schneider Children’s Medical Center, Petah-Tikva, Israel between January 2001 and December 2009. Mean age 16.1±9.7 years; 78 males (55%) and 64 (45%) females. Sources of referral were: (1) Individuals with FISH confirmed diagnosis of 22q11.2DS referred by clinical genetics departments in Israel; (2) Individuals clinically suspected of the presence of 22q11.2DS, referred by cardiologists, developmental pediatrics, psychiatrists and clinicians at a cleft palate unit.

The study protocol was approved by the Rabin Medical Center Review Board, Petah-Tikva, Israel and written informed consent was obtained from the study participants and their parents or guardians.

### Clinical evaluation

Study subjects and their parents underwent a comprehensive medical and developmental intake with a structured clinical checklist composed of the comprehensive multisystem possible clinical symptoms of 22q11.2DS and developmental history [2]. Subjects were referred to further medical evaluations that included cardiac check-up (including electrocardiography and echocardiography), assessment for the presence of typical facial features, evaluation of cleft anomalies (including multi-video fluoroscopy and video endoscopy) and blood calcium levels as previously described [13].

### Neuropsychiatric evaluation

All subjects and their parents were interviewed by skilled clinicians, who were trained until they achieved satisfactory inter-rater reliability scores with the senior investigator (D.G.), using the Hebrew version of the Schedule for Affective Disorders and Schizophrenia for School-Aged Children, Present and Lifetime (K-SADS-PL) [14,15]. Adults and their parents (when available) were interviewed with the Structured Clinical Interview for Axis I DSM-IV Disorders (SCID) [16]. The ADHD module from the K-SADS was added to the SCID to evaluate the presence of ADHD.

Cognitive evaluation was conducted with the age-appropriate versions of the Wechsler Intelligence Scale [17,18].

### Laboratory techniques

FISH analysis for 22q11.2 deletion was performed according to standard procedures in the cytogenetic laboratory at the Raphael Recanati Genetics Institute, Rabin Medical Center, Israel, using the LSI TUPLE1 (HIRA) or N25 commercial probes (22q11.2) and the LSI ARSA as a sub-telomeric 22q13.3 control probe (Vysis Inc., Downers Grove, IL, USA).

### DNA extraction

DNA from venous blood was extracted using a commercial kit (Promega Corporation, Madison, WI, USA). All DNA samples were quantified by NanoDrop ND 1000 Spectrophotometer (NanoDrop Technologies, City, USA) and stored at −70°C.

### MLPA analysis

MLPA is a PCR- based method designed to detect gene dosage abnormality by relative quantifications. The SALSA MLPA P250-A1 DiGeorge kit (MRC-Holland, Amsterdam, Netherlands) was used to identify copy number variations (CNVs) in the 22q11.2 region (30 probes) and in other chromosomal regions (4q35, 8p23, 9q34, 10p15, 17p13; 18 probes) according to manufacturer's instructions ( http://www.mrc-holland.com). PCR amplification products were separated by capillary electrophoresis using ABI-Prism 3100 Genetic Analyzer (Applied Biosystems, Foster City, CA) at the Hy Laboratories Ltd., Rehovot, Israel. The data were analyzed using the Coffalyser VBA analysis software V8 ( http://www.mlpa.com/coffalyser).

## Results

### Molecular analyses

**Figure 1:**
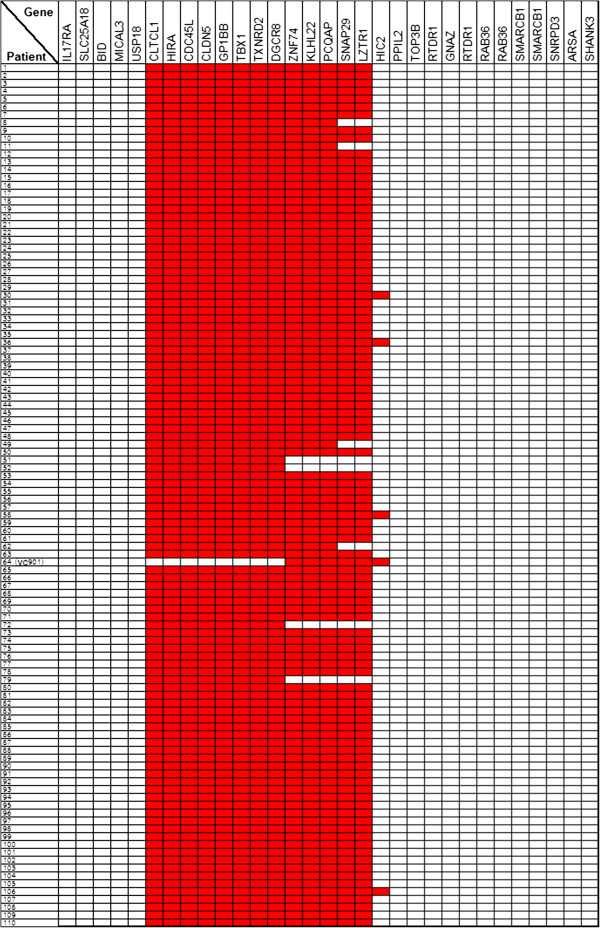
**MLPA results for 110 individuals with 22q11.2 deletion.** Shown below are 30 gene probes within 22q11.2 region (MLPA-P250 kit) which were used for identifying the copy number variation. The copy number is indicated by color: red- one copy (hemizygous deletion); white- two copies.

Patients were tested for a deletion in 22q11.2 by the FISH technique as part of their routine workup upon admission to the BNC in order to establish the diagnosis of 22q11.2DS. The 22q11.2 deletion was detected in 109 of 140 subjects (77.8%) using either TUPLE1 (HIRA) or N25 commercial probes. The MLPA test was performed on all patients admitted to the BNC with suspected diagnosis of 22q11.2DS, irrespective of their FISH status, as a confirmatory test to FISH and as a tool for determining the deletion location and boundaries. Using the MLPA P250-A1 DiGeorge kit, a 22q11.2 deletion was detected in 110 (77.5%) of the 142 individuals examined. Duplications were not detected in any of the subjects. Additional probes outside 22q11.2 contained in this kit (from chromosomes 4q34, 8p23, 9q34.3, 10p15, 17p13.3 and 22q13) did not reveal additional CNVs (either deletions or duplications), using a criterion of 'more than one consecutive probe showing significant deviation from the normal values'. A detailed graphical representation of the MLPA analysis for 110 individuals with deletions is depicted in Figure 1. Most subjects (97/110; 88.2%) showed a similar pattern of deletion spanning molecular probes from *CLTCL1* to *LZTR1* representing the 3Mb TDR.

**Figure 2:**
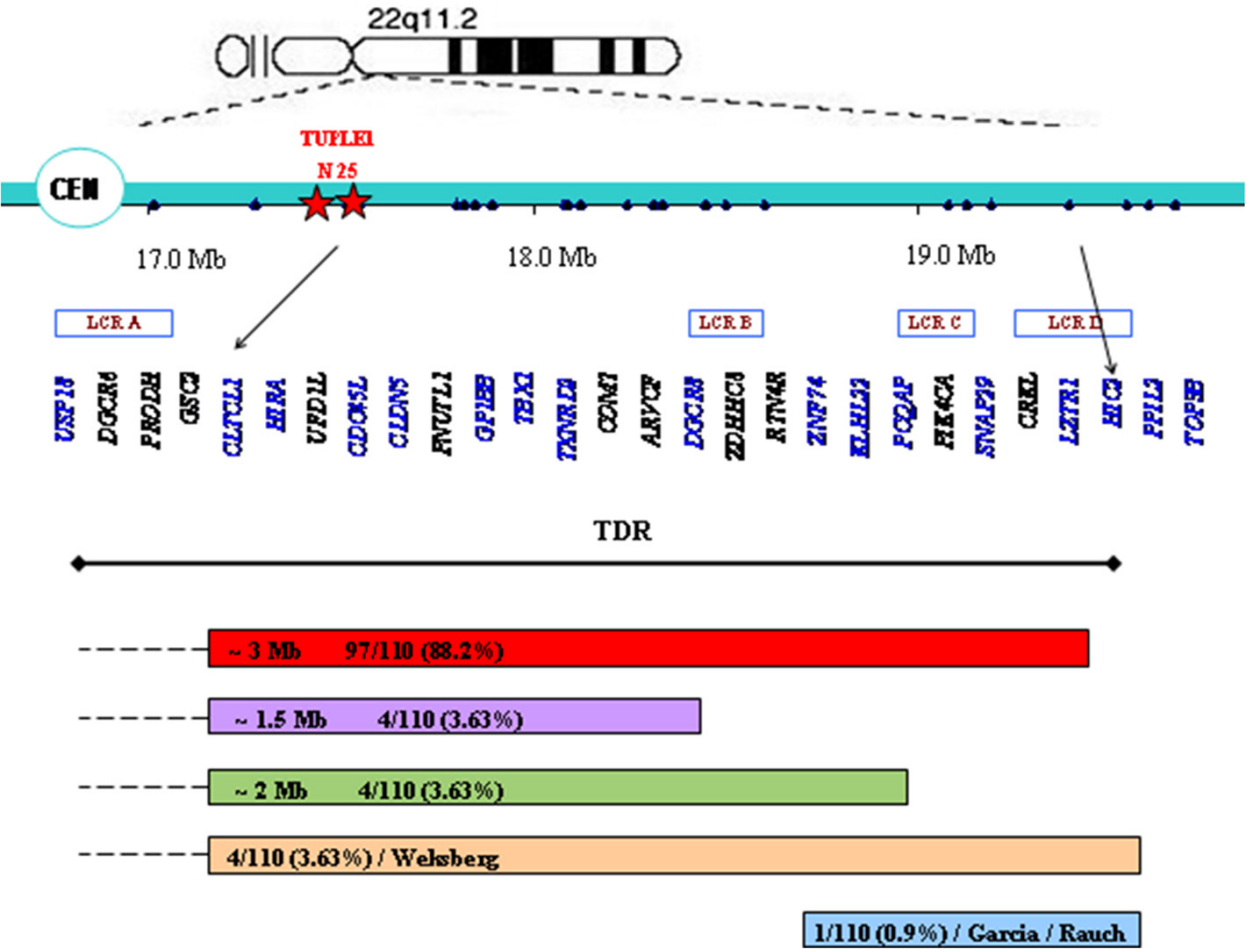
**Schematic representation of 22q11.2 deletions detected by MLPA.** Genes marked in blue contain MLPA P250-A1 probes. A typically deleted region (TDR) is indicated in red; non-TDR deletions are presented by other colors. FISH probes – N 25 and TUPLE1 (*HIRA*) are indicated by stars.

The deletions in 13 individuals (11.8%) differed from TDR and thus were referred to as non-TDR deletions. Four subjects had a 1.5Mb deletion located between *CLTCL1* and *DGCR8* probes (LCR A-B), four other subjects had a deletion between *CLTCL1* and *PCQAP* (LCR A-C) and a larger deletion extending from *CLTCL1* to *HIC2* (LCR A-D+) was identified in 4 individuals (Figure 2). One individual (VC901) carried a short (~1Mb) deletion between LCR B and D+.

There was high agreement between the FISH and MLPA results. All patients identified by FISH (N=109) were confirmed by MLPA, but MLPA identified an additional individual with a 22q11.2 deletion (VC901, Figure 1, 2). This deletion could not be identified by FISH since its starting point is distal to the FISH N25 and TUPLE1 probes (Figure 2). All individuals that were found to be non-deleted by MLPA (N=30) were also FISH negative.

Since the P-250 MLPA kit lacks sufficient probes in the proximal end of the deletion we used the RT qPCR technique as a supplementary method. We studied the deletion status in the proximal breakpoint using SYBR Green labeled *PRODH* probe and the *VPREB1* probe as a control [11]. It was found that the *PRODH* gene was hemizygously deleted in most (68/71; 95.8%) subjects tested. Out of 3 deletions that did not contain the *PRODH* gene two were LCR A-D (~3Mb) and one was LCR A-B (1.5Mb).

### Genotype-phenotype analysis

Tables 1 and 2 summarize the cumulative physical and psychiatric data of the 110 subjects with 22q11.2 deletions grouped according to the type of deletion. There are 97 individuals with TDR and 13 individuals divided into four groups of different types of non-TDR deletions. The small number of individuals in each of the different non-TDR deletions and the inherent variability of the clinical data preclude rigorous statistical analysis. However, it can be seen that there are no large differences between the groups in terms of physical and psychiatric manifestations typical to the 22q11.2DS. It is of note that hypocalcemia was present in 27.7% (28/101) of individuals with 3Mb and larger deletions and in none (0/8) of the individuals with the smaller proximal deletions (1.5 and 2Mb) (Table 1).

**Table 1:** Clinical data for 22q11.2DS subjects - Physical features

**Deletion type**	**Cardiovascular anomalies**	**Palate abnormality**	**Facial dysmorphology**	**Hypocalcemia**
LCR A-D (TDR)	67/97(0.69)	80/97 (0.82)	85/87 (0.98)	27/97 (0.28)
LCR A-B (~1.5 Mb)	1/4 (0.25)	3/4 (0.75)	4/4 (1.00)	0
LCR A-C (~2 Mb)	3/4 (0.75)	3/4 (0.75)	4/4 (1.00)	0
LCR A-D+	4/4 (1.00)	4/4 (1.00)	4/4 (1.00)	1/4 (0.25)
LCR B-D+	1/1 (1.00)	1/1 (1.00)	0	0

**Table 2:** Clinical data for 22q11.2DS subjects - Psychiatric and cognitive features

**Deletion**	**Age [years] Mean ±SD (N)**	**VIQ**	**PIQ**	**FIQ**	**Psychosis**			**ADHD**	**MDD**	**Anxiety disorders**	**OCD**	**ASDs**
		**Mean ±SD (N)**			**SZ**	**SZaff**	**Psychotic dis. NOS**					
LCR A-D 3Mb (TDR)	17.39±10.1 (97)	77.5±12.6 (81)	76.2±13.6 (80)	74.2±12.5 (82)	7/80 (0.09)	1/80 (0.01)	3/80 (0.04)	36/80 (0.45)	8/80 (0.10)	32/80 (0.40)	8/80 (0.10)	3/69 (0.04)
LCR A-B (~1.5 Mb)	20.50±17.41 (4)	81.0+18.0 (3)	73.0±14.0 (3)	75.0+16.8 (3)	0	1/4 (0.25)	0	2/4 (0.50)	0	1/4 (0.25)	0	0
LCR A-C (~2 Mb)	17.00±15.56 (4)	79.7+8.5 (3)	70.0±2.0 (3)	68.8±6.5 (4)	0	0	0	3/4 (0.75)	0	2/4 (50)	0	0
LCR A-D+	11.25±2.63 (4)	79.3+11.4 (4)	75.3±12.3 (4)	75.0±10.5 (4)	0	0	0	4/4 (1.00)	1/4 (0.25)	2/4 (0.50)	1/4 (0.25)	0
LCR B-D+	20	69	55	59	0	0	1	1/1 (1.00)	0	1/1 (1.00)	0	0

*VIQ*, *PIQ* and *FIQ* – verbal, performance and full IQ, respectively, *SZ* - Schizophrenia, *SZaff* - Schizoaffective Disorder, Psychotic dis. *(NOS)* - Psychotic Disorder Not Otherwise Specified, *ADHD* - Attention Deficit Hyperactivity Disorder, *MDD*- Major Depressive Disorder, *OCD* – Obsessive Compulsive Disorder, *ASDs* – autism spectrum disorders.

Table 3 gives a detailed description of the clinical data of each the 13 non-TDR subjects. Individuals with the 1.5Mb deletion (LCR A-B) exhibited fewer cardiovascular anomalies compared to the other non-TDR subjects (1/4, 25% vs. 8/9, 88.9%; p=0.052).

**Table 3:** Clinical data of 22q11.2DS subjects with non-TDR deletion

**ID**	**Age at diagnosis [years]**	**Deletion**	**Psychiatric diagnoses**	**Cardiovascular anomalies**	**Facial dysmorphology**	**Palate abnormality**
VC671	16	LCR A-B (~1.5 Mb)	MDD GAD	No	Eyes, ears, chin, nose	Cleft palate
VC672	46	LCR A-B (~1.5 Mb)	Schizoaffective disorder	No	Chin	No
VC1081	7	LCR A-B (~1.5 Mb)	ADHD	No	Mouth, ears, chin, nose, face	Occult submucous cleft palate
VC1161	13	LCR A-B (~1.5 Mb)	ADHD	PDA, bicuspid aortic valve	Eyes, mouth, ears, chin, nose, face	Hypernasal speech
VC171	40	LCR A-C (~2 Mb)	MDD, Specific phobia, PTSD	PFO, hemitruncus atresia of Rt pulmonary, high origin of Lt pulmonary artery from ascending aorta with big Lt to Rt shunt.	Eyes, mouth, ears, nose, face	No
VC201	13	LCR A-C (~2 Mb)	ADHD, Specific phobia	Rt aortic arch with aberrant left subclavian. Vascular ring - diagnosed at birth.	Eyes, mouth, ears, nose, face	Occult submucous cleft palate
VC641	7	LCR A-C (~2 Mb)	ADHD	VSD, ASD - spontaneous closure, aplastic Rt carotis and medial Lt upper carotis	Ears	Occult submucous cleft palate
VC871	8	LCR A-C (~2 Mb)	ADHD	No	Eyes, ears, nose, face	Occult submucous cleft palate
VC431	10	LCR A-D+	ADHD, OCD	Small VSD- spontaneously closed. Aberrant Rt. subclavia from descendent aorta	Eyes, mouth, ears, chin, nose, face	Occult submucous cleft palate
VC511	9	LCR A-D+	ADHD, Specific phobia	Two small VSDs	Eyes, mouth, ears, chin, nose	Occult submucous cleft palate
VC741	11	LCR A-D+	ADHD	ASD spontaneously closed, abnormal aortic valve.	Eyes, mouth, ears, chin, nose, face	Occult submucous cleft palate
VC1621	15	LCR A-D+	ADHD, MDD	Bicuspid aortic valve	Eyes, mouth, face	Occult submucous cleft palate
VC901	20	LCR B-D+	Psychotic dis. (NOS), ADHD, GAD, SA, Specific and Social phobia	VSD	Eyes, mouth, chin, nose, face	Cleft lip and palate

*ADHD*- Attention Deficit Hyperactivity Disorder, *GAD*- Generalized Anxiety Disorder, *MDD*- Major Depressive Disorder, *PTSD*- Posttraumatic Stress Disorder, *VSD*- Ventricular Septal Defect, *ASD*- Atrial Septal Defect, *PDA*- Patent Ductus Arteriosus, *PFO*- Patent Foramen Ovale *SA*- Separation Anxiety Disorder, Psychotic dis. *(NOS)* - Psychotic Disorder Not Otherwise Specified.

### Case report

**Figure 3:**
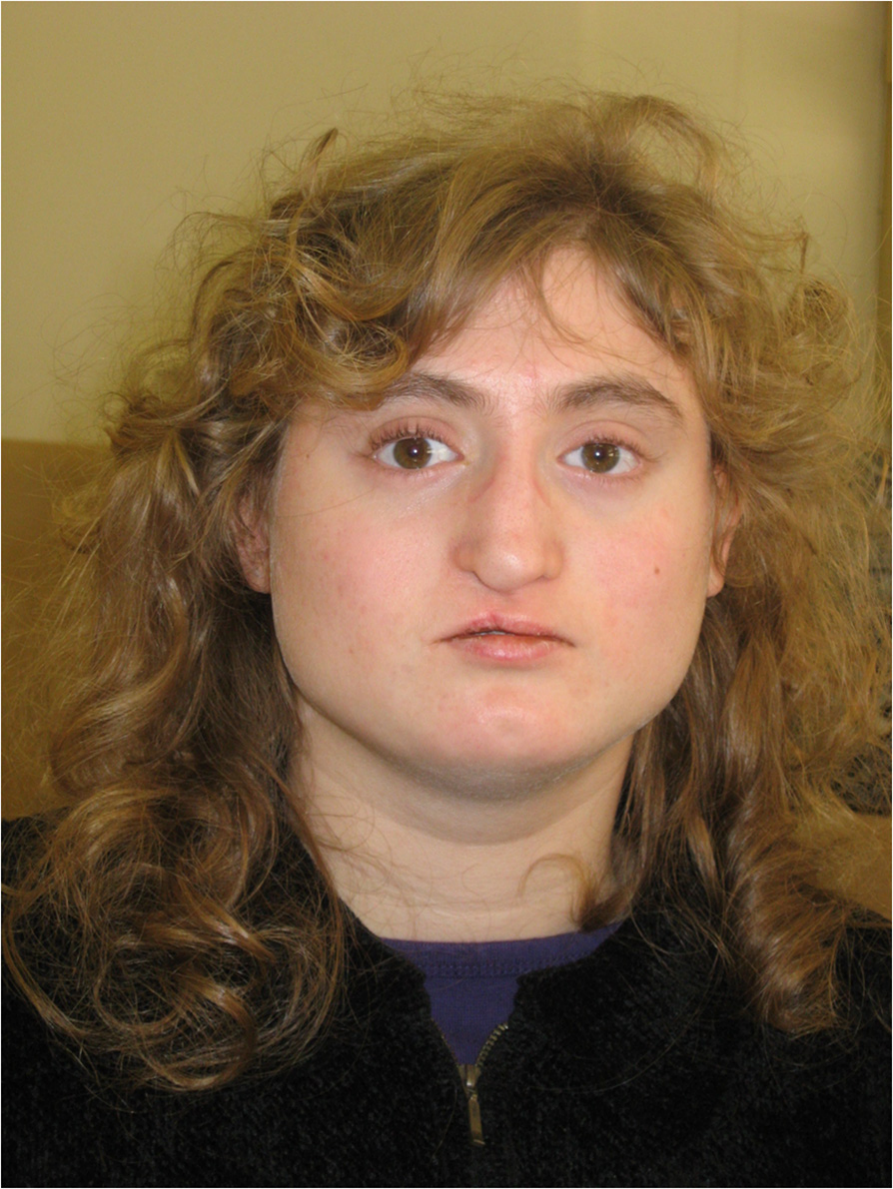
**Facial appearance of Patient VC901 with atypical 22q11.2 deletion.** Note the broad square face, hypotelorism, narrow nasal base and broad nasal bridge, overhanging nasal tip, deviated nasal tip (state post cleft lip repair), short philtrum, narrow mouth, chin dimple and broad neck.

Patient VC901 carried a distal LCR B-D+ deletion located between *ZNF74* and *HIC2* probes (Figure 2). We elaborate below on the clinical features of this patient because she carried a rare deletion with only two other similar cases in the literature [19,20] and may allow studying the impact of the deleted genes in this patient on its particular phenotype. Patient VC901 was referred to our clinic at the age of 10 years as a suspected 22q11.2DS by a trained speech therapist based on her physical and neuropsychiatric profile. FISH analysis using the N25 probe failed to detect a deletion in this individual. Patient VC901 was born with ventricular septal defect (VSD) that was corrected by surgery, cleft lip and palate, retrognathia, hypernasal speech, and short stature (below the third percentile for height, weight and head circumference). The facial features share some similarities with 22q11.2DS (see Figure 3). She has a broad square face, hypotelorism, narrow nasal base and broad nasal bridge, overhanging nasal tip, deviated nasal tip (state post cleft lip repair), short philtrum, narrow mouth, chin dimple and broad neck. Already at the age of 5 years she was diagnosed with dysgraphia and dyscalculia. Results on Wechsler testing at the age of 10 years were: full scale IQ 59 (verbal IQ 69, performance IQ 55). Psychiatric assessment at the age 13 years conducted by K-SADS indicated that Patient VC901 is suffering from severe anxiety disorders including generalized and separation anxiety disorders and specific and social phobias. In addition Patient VC901 was coping with ADHD, predominantly inattentive type. She also exhibited bizarre behaviors which were diagnosed as subthreshold psychotic symptoms. On second evaluation at the age of 20 years Patient VC901 was diagnosed with psychotic disorder not otherwise specified, marked by delusions of reference and recurrent episodes in which she threatened to stab her parents with a knife, with a Positive and Negative Syndrome Scale (PANSS) score of 89. Her cognitive performance showed some improvement compared to the first evaluation with full scale IQ score 67 (verbal IQ 73, performance IQ 65).

### Individuals with no detected deletion

In 32 (22.5%) of 142 subjects referred to the BNC with suspicion of 22q11.2DS no deletions were detected either by MLPA or by FISH. To illustrate the problematic diagnosis of subjects showing some 22q11.2DS-like phenotype but with no apparent deletion we selected 12 individuals with the most pronounced clinical manifestations typical to 22q11.2DS and present them in Table 4.

**Table 4:** Clinical data of individuals without 22q11.2 deletion

**ID**	**Age**	**Gender**	**Palate abnormality**	**Cardiovascular anomalies**	**Facial dysmorphology**	**Hypocalcemia**
VC411	11	F	Occult submucous cleft palate	VSD	Eyes, chin, face	No
VC561	19	F	Occult submucous cleft palate	TOF	Eyes, mouth, nose, face	No
VC611	19	M	Occult submucous cleft palate	Aortic stenosis	Eyes, mouth, face	Yes
VC631	14	F	Occult submucous cleft palate	VSD	Eyes, mouth, chin, face	No
VC761	24	F	Submucous cleft palate	PDA	Eyes, mouth, nose, face	No
VC861	13	M	Cleft palate	VSD, hypoplastic left ventricle	Eyes, nose, face	No
VC941	5	M	Velopharyngeal insufficiency	VSD	Mouth	No
VC951	10	M	Hypernasal speech	VSD	Nose, face	No
VC1021	23	F	Occult submucous cleft palate	Truncus arteriosus	Eyes, mouth, ears, chin	Yes
VC1031	11	M	Hypernasal speech	ASD	Mouth, nose, face	No
VC1201	15	F	Cleft palate	Aortic stenosis	Face	No
VC1464	7	F	Palate insufficiency	PFO, Pulmonary stenosis	Mouth, chin, nose	Yes

*VSD*- Ventricular septal defect, *ASD*- Atrial septal defect, *TOF* - Tetralogy of Fallot, *PDA*- Patent ductus arteriosus, *PFO*- Patent foramen ovale.

Interestingly, it appears that the variety and severity of the clinical symptoms in these subjects are very similar to those found in confirmed 22q11.2DS patients.

## Discussion

Analyzing a relatively large 22q11.2DS cohort allowed us to compare the FISH and MLPA methods, perform molecular analysis of the 22q11.2 deletion in our patients and examine the phenotype-genotype relationship.

The MLPA technique confirmed all the deletions that were previously detected by FISH in our patients while the FISH assay failed to detect one atypical deletion because it was outside the location of the FISH probes. Thus the MLPA is clearly superior to FISH as a diagnostic tool and a useful method for deletion characterization, essential for genotype-phenotype analysis. CGH and SNP array techniques can also detect atypical deletions but their costs are substantially higher. Other studies have recommended using MLPA as a diagnostic tool [8,21]. However, MLPA is limited in identifying the deletion boundary that is determined by the availability of probes in the deletion breakpoints. The MLPA kit that we used (P250-A1) lacked probes in the candidate *PRODH* gene located in the proximal breakpoint which was found to be associated with psychotic disorders in 22q11.2DS [22,23]. To solve this problem we used qPCR technique and found that the *PRODH* gene was deleted in most (94.4%) of the 22q11.2DS individuals, as was found previously [6,24]. The MLPA kit released after we performed our study (P324-A2) already contained several *PRODH* gene probes.

### Molecular characteristics of the 22q11.2 deletion

We identified 5 types of 22q11.2 deletions. According to the results of the specific MLPA kit used, the proximal starting point for all deletions, except one, was located in the *CLTCL1* gene probe, near LCR A (Figure 2). The distal breakpoints of the deletions were located in LCRs B, C, D and D+ thus determining the size and location of the deletions. Ninety seven out of 110 individuals (88.2%) carried the typical ~3Mb deletion (LCR A-D) consistent with frequencies found in other studies (77–88.7%) [20,21,24-27].

The largest deletion (LCR A-D+), a variant of the 3Mb deletions, was present in 3.63% of the subjects compared to 4.5-6.5% in previous reports [8,24]. The prevalence of the proximal deletions of 1.5Mb (LCR A-B) and 2Mb (LCR A-C), each 3.63% in our subjects, varied largely in other studies [20,21,24-27], probably due to different sample sizes and resolution of the mapping methods used.

### Genotype-phenotype relationship

Studying the genotype-phenotype relationship in 22q11.2DS is a formidable task. The difficulties are obvious: there are up to 200 possible clinical symptoms [4], the clinical variability is large even in individuals with the same type of deletion and the statistical power is low because most individuals harbor the common 3Mb TDR and only a few with other entities. Yet, it can potentially shed light on the role of the genes that contribute to the physical and psychiatric symptoms expressed in this disorder. The quantitative comparison between 97 individuals with TDR and those with four different types of nonTDR deletions along several physical and neuropsychiatric-cognitive parameters did not reveal statistically significant differences. This could be due to methodological limitations such as sample size, and the complexity of the genetic and epigenetic mechanisms governing the phenotype in 22q11.2DS. Our results are supported by most previous studies that could not demonstrate a correlation between the size and the location of the deletion and the clinical features of the subjects [24,25,28-30]. However, Rauch et al. (2005) demonstrated a correlation between deletion characteristics and phenotypic expression by assessment of individuals with typical and atypical 22q11.2 deletions, showing that atypical CHD and mild dysmorphism are recognizable feature of atypical distal deletions [20]. Recently, a case-report of monozygotic twins differing in their deletion size and clinical expression was published, indicating a possible role of deletion characteristics for the phenotype [31].

In accordance with previous studies [32] hypocalcemia was found in 27.7% of patients with large deletions (3Mb, LCR A-D and LCR A-D+) but was absent in those carrying the proximal nested deletions (1.5 and 2Mb). Interestingly, neither was it observed in patients with distal nested deletions [33]. Although the number of cases is small, this observation may suggest that haploinsufficiency of genes both at the proximal and the distal part of 22q11.2 is required in order to cause hypocalcemia.

The presented clinical data of each individual carrying a non-TDR deletion are not in themselves informative but may be of help in the effort to build a common data base. More informed and educated conclusions could be made on this intriguing question by applying unified molecular methods for mapping the 22q11.2 deletion (high resolution methods such as CGH and SNP arrays and next generation sequencing) and using agreed clinical parameters and scoring system for characterizing the phenotype.

### Individuals without 22q11.2 deletion

Before the era of molecular diagnosis patients were diagnosed as VCFS (22q11.2DS) solely based on their clinical symptoms. When molecular genetics methods were introduced it turned out that in about 20% of the patients classified as VCFS no deletion could be detected [20,21] and the question of the etiology of their phenotype is still unknown. Interestingly, when we examined a subgroup of these individuals it appeared that the variety and severity of clinical symptoms in these subjects are very similar to those found in confirmed 22q11.2DS patients. Subjects may have been misdiagnosed because they had a combination of relatively common symptoms, such as cardiac anomalies and neuropsychiatric disorders, that when considered together phenocopy the 22q11.2DS. It is also possible that these patients do carry very small deletions or even point mutations which are below the resolution of the methods used or they have other microdeletion or microduplication syndromes.

### The case with atypical nested distal deletion

Patient VC901 carries a rare, small deletion that does not include the nested proximal 1.5Mb region which harbors several important candidate genes, thought to be sufficient for producing the syndrome [34]. This case portrays the complexity of the molecular mechanisms controlling the 22q11.2DS phenotype, indicating the possibility of redundancy in causative genes. Since the "classical" candidate genes are not deleted in this person other candidate genes residing in the nested distal region may be responsible for the physical features and neuropsychiatric manifestations. For example, of the candidate genes that have been shown to be involved in cardiac malformations (*HIRA*, *UFD1L, TBX1* and *CRKL*) [35-39]*CRKL* is one that is located in the distal deletion region and has been found to affect cardiac neural crest derivatives in mice [36]. Its loss may be the causative event for the CHD in this case. In a similar manner, the neuropsychiatric phenotype may be affected by the deletion of *PIK4CA* and *SNAP29* from the distal region which have been demonstrated to be associated with schizophrenia [40-43], and not by the putative schizophrenia genes *PRODH*, *COMT*, *DGCR8* and *ZDHHC8*. Another genetic mechanism that may affect the phenotype involves control elements in the deleted region that act by *cis* mechanisms on the expression of the non-deleted candidate genes as in the dosage-sensitive interaction between *Tbx1*and *Crkl* in mice [44].

The longitudinal neuropsychiatric follow-up of this patient has raised the intriguing question of whether multiple genes in the 22q11.2 region are responsible for the emergence of schizophrenia in 22q11.2DS patients. Her psychiatric developmental trajectory is typical of 22q11.2DS namely, anxiety and subthreshold positive psychotic symptoms during childhood evolving to a full psychotic disorder in adulthood. Yet her cognitive developmental trajectory is not typical, as her IQ scores did not decline and even improved a little during young adulthood, whereas in typical 22q11.2DS patients there is a cognitive decline especially of verbal IQ [45, 46]. This might suggest that the neuropsychiatric manifestations in 22q11.2DS are governed by several sets of genetic elements. It is tempting to speculate that genes such as *PIK4CA* and *SNAP29* that are deleted in our patient may be involved in the emergence of psychotic "positive symptoms" while others, such as *PRODH* and *COMT* that are not deleted, play a role in the cognitive decline process.

Two other 22q11.2DS cases with similar distally nested deletion have been reported but they are not readily comparable to the present case because of the probands' young age. One was a patient with TOF and facial dysmorphology, last assessed at 9 months of age [19], and the other a 6 year-old with typical facial features, significant developmental delay and increased anxiety suggestive of possible early signs of psychiatric illness [20].

## Conclusions

The MLPA method was accurate and efficient in diagnosing 22q11.2DS in our patients and in defining ambiguous cases. In our opinion, for a mid-size laboratory it represents the best choice as an intermediary technique between the classical FISH method and the high throughput methods such as CGH, SNP arrays and deep sequencing.

Although the quantitative genotype-phenotype correlations in our cohort were not statistically significant, the clinical data presented may be added to the body of evidence continually accumulating by interested scientists. Individuals with unique deletions may shed light on specific issues as in the case of patient VC901. Her case suggests that there is redundancy of genes causing physical and neuropsychiatric phenotype in 22q11.2DS and that the psychiatric and cognitive trajectories may be governed by different genes.

We believe that the study of the genotype-phenotype correlation would be best conducted using a comprehensive approach of creating a multicenter consortium that will establish a large data base of patients to be characterized by uniform clinical standards and by state-of-the art molecular methods.

## Abbreviations

22q11.2DS: 22q11.2 deletion syndrome; ADHD: Attention deficit hyperactivity disorder; ASD: Atrial septal defect; ASDs: Autism spectrum disorders; BNC: Behavioral Neurogenetics Center; CGH: Comparative genomic hybridization; CHD: Congenital heart defects; CNVs: Copy number variations; FISH: Fluorescent *in situ* hybridization; FIQ: Full IQ; GAD: Generalized anxiety disorder; LCR: Low copy repeats; MDD: Major depressive disorder; MLPA: Multiplex ligation probe amplification; OCD: Obsessive compulsive disorder; PANSS: Positive and negative syndrome scale; PDA: Patent ductus arteriosus; PFO: Patent foramen ovale; PIQ: Performance IQ; Psychotic dis. (NOS): Psychotic disorder not otherwise specified; PTSD: Posttraumatic stress disorder; qPCR: Quantitative polymerase chain reaction; SA: Separation anxiety disorder; SNP: Single nucleotide polymorphism; SZ: Schizophrenia; SZaff: Schizoaffective disorder; TDR: Typically deleted region; TOF: Tetralogy of Fallot; VCFS: Velocardiofacial syndrome; VIQ: Verbal IQ; VSD: Ventricular septal defect.

## Competing interests

The authors declare that they have no competing interests.

## Authors’ contributions

EM performed laboratory work including MLPA and qPCR analyses, data analyses and interpretation and initiated the draft of the manuscript. AF participated in designing the study and drafting the manuscript. MC and MP participated in laboratory work including DNA extraction and MLPA analysis and results interpretation. OZ and TG participated in providing clinical evaluations. LBV carried out the clinical characterization of patient VC901. AW participated in designing the study, data analyses and participated in drafting the manuscript. DG recruited and clinically followed the patients**,** carried out clinical evaluations and follow-up and participated in designing the study and drafting the manuscript. All autors read and approved the final manuscript.

## Pre-publication history

The pre-publication history for this paper can be accessed here:

http://www.biomedcentral.com/1471-2350/13/122/prepub
